# Autism risk gene *Cul3* alters neuronal morphology via caspase-3 activity in mouse hippocampal neurons

**DOI:** 10.3389/fncel.2024.1320784

**Published:** 2024-05-09

**Authors:** Qiang-qiang Xia, Anju Singh, Jing Wang, Zhong Xin Xuan, Jeffrey D. Singer, Craig M. Powell

**Affiliations:** ^1^Department of Neurobiology, Marnix E. Heersink School of Medicine & Civitan International Research Center, University of Alabama at Birmingham, Birmingham, AL, United States; ^2^Department of Biology, Portland State University, Portland, OR, United States

**Keywords:** cullin-3, dendrite, axon, spine, synapse, caspase-3

## Abstract

Autism Spectrum Disorders (ASDs) are neurodevelopmental disorders (NDDs) in which children display differences in social interaction/communication and repetitive stereotyped behaviors along with variable associated features. *Cul3*, a gene linked to ASD, encodes CUL3 (CULLIN-3), a protein that serves as a key component of a ubiquitin ligase complex with unclear function in neurons. *Cul3* homozygous deletion in mice is embryonic lethal; thus, we examine the role of *Cul3* deletion in early synapse development and neuronal morphology in hippocampal primary neuronal cultures. Homozygous deletion of *Cul3* significantly decreased dendritic complexity and dendritic length, as well as axon formation. Synaptic spine density significantly increased, mainly in thin and stubby spines along with decreased average spine volume in *Cul3* knockouts. Both heterozygous and homozygous knockout of *Cul3* caused significant reductions in the density and colocalization of gephyrin/vGAT puncta, providing evidence of decreased inhibitory synapse number, while excitatory synaptic puncta vGulT1/PSD95 density remained unchanged. Based on previous studies implicating elevated caspase-3 after *Cul3* deletion, we demonstrated increased caspase-3 in our neuronal cultures and decreased neuronal cell viability. We then examined the efficacy of the caspase-3 inhibitor Z-DEVD-FMK to rescue the decrease in neuronal cell viability, demonstrating reversal of the cell viability phenotype with caspase-3 inhibition. Studies have also implicated caspase-3 in neuronal morphological changes. We found that caspase-3 inhibition largely reversed the dendrite, axon, and spine morphological changes along with the inhibitory synaptic puncta changes. Overall, these data provide additional evidence that *Cul3* regulates the formation or maintenance of cell morphology, GABAergic synaptic puncta, and neuronal viability in developing hippocampal neurons in culture.

## Graphical abstract

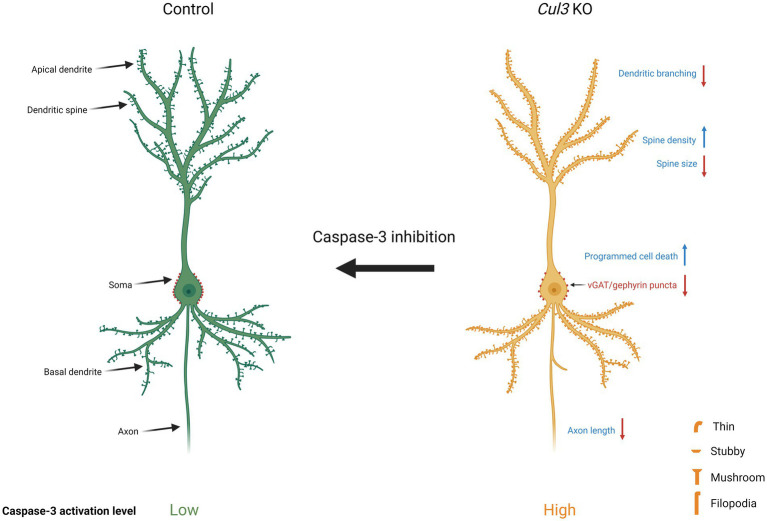

## Introduction

Autism spectrum disorders (ASDs) are common, sometimes debilitating disorders affecting social interaction/communication and repetitive behaviors (American Psychiatric Association, 2000; Lord et al., 2018). Approximately 15–20% of ASDs are caused by identifiable genetic mutations (Abrahams and Geschwind, 2010; Iossifov et al., 2014; Geschwind and State, 2015). Ubiquitin-proteasome degradation pathways are strongly implicated in ASD/NDD (Elgersma, 2015; Yi et al., 2015) with potential therapeutic approaches discovered (Huang et al., 2012). The *Cul3* gene, coding for CUL3, a scaffolding component of an E3 ubiquitin ligase complex, is among several high-confidence ASD genes in exome sequencing studies focused on gene-deleterious, loss-of-function mutations (O'Roak et al., 2011, 2012a; De Rubeis et al., 2014; Iossifov et al., 2014; Geschwind and State, 2015; Sanders et al., 2015), making it of interest in understanding ASD pathophysiology.

Many susceptibility genes for autism are involved in regulating synapse formation, maintenance, or function (Kwon et al., 2006; Tabuchi et al., 2007; Blundell et al., 2010; Kouser et al., 2013; De Rubeis et al., 2014; Iossifov et al., 2014; Geschwind and State, 2015; Sanders et al., 2015; Araujo et al., 2017; Escamilla et al., 2017). Although little is known about the role of *Cul3* in mammalian brain, deletion of the gene *Kctd13*, a gene in the 16p11.2 recurrent CNV region (Kumar et al., 2008; Marshall et al., 2008; Weiss et al., 2008; Bijlsma et al., 2009; McCarthy et al., 2009; Fernandez et al., 2010; Shinawi et al., 2010; Zufferey et al., 2012; Steinberg et al., 2014; D'Angelo et al., 2016) and a binding partner of CUL3, leads to decreased synaptic transmission and decreased synapse numbers in the hippocampus and other brain regions (Escamilla et al., 2017). Unlike *Kctd13* homozygous deletion, however, deletion of *Cul3* does not result in viable homozygous offspring (Singer et al., 1999) [wild-type (WT): 40.3%, heterozygous (Het): 59.7%, homozygous (Hom): 0 in 206 mice], indicating very different phenotypic effects in spite of some mechanistic overlap.

Previous studies indicate complex results of *Cul3* heterozygous deletion in mouse brain morphology. *Cul3* heterozygous deletion does not cause major neuronal morphology abnormalities except slightly decreased dendritic complexity (Rapanelli et al., 2019; Morandell et al., 2021; Xia et al., 2023), while another study reports decreased dendritic branching in primary cortical neurons (Amar et al., 2021). *Cul3* heterozygous deletion decreases spine density in prefrontal cortical neurons (Rapanelli et al., 2019) but increases apical spine density in hippocampal CA1 pyramidal neurons (Dong et al., 2020). Loss of *Cul3* also results in dysregulation of cytoskeletal proteins and impacts cytoskeletal dynamics (Amar et al., 2021; Morandell et al., 2021). These inconsistent results may be related to the different genetic backgrounds, models, or incompleteness/mosaicism of the deletion, which makes *Cul3*’s function in neurons, particularly during early development, unclear.

In addition, *in-situ* hybridization (ISH) data from the Allen Brain Atlas showed significantly high CUL3 expression in the pyramidal and granule cell layers of CA1 and DG of the hippocampus (Lin et al., 2023), and the hippocampus is important for several functions that are disrupted in ASD, e.g., social interaction (Hitti and Siegelbaum, 2014; Rubin et al., 2014; Tavares et al., 2015; Meira et al., 2018; Schafer and Schiller, 2018), spatial reasoning (Doeller et al., 2010; Jacobs et al., 2013; Lee et al., 2013; Brown et al., 2014) and memory (Fortin et al., 2002; Bartsch et al., 2011; Cooper et al., 2017; Cooper and Simons, 2019). Individuals with ASD may have abnormal hippocampal structure and function (Kemper and Bauman, 1993; Raymond et al., 1996; Bailey et al., 1998; Lawrence et al., 2010; Wegiel et al., 2010; Varghese et al., 2017). ASD symptoms typically become increasingly apparent when the hippocampus reaches major developmental milestones at around 2 years old (Banker et al., 2021). Thus, to understand how complete, homozygous deletion of *Cul3* might affect early neuronal development, we created primary cultures of hippocampal neurons from postnatal day 1 (P1) brains and used adeno-associated virus (AAV) transduction to express either Cre-GFP (cre recombinase and green fluorescent protein) or GFP alone in WT, Het, and Hom f*Cul3* developing hippocampal neurons *in vitro*.

After confirming expected deletion of *Cul3* in AAV-Cre-GFP-treated neurons, we identified axon, dendrite and spine morphological changes in *Cul3* mutant neurons. Also, we examined the density and colocalization of pre- and post-synaptic excitatory/inhibitory synapse markers. Previous studies implicated elevated caspase-3 after *Cul3* deletion (Kaplan et al., 2010; Amar et al., 2021; Morandell et al., 2021), and caspase-3 is known to modulate apoptosis (Wang and Lenardo, 2000; D'Amelio et al., 2012). Therefore we also confirmed elevated caspase-3 in our *Cul3* deletion neurons and used caspase-3 inhibitor Z-DEVD-FMK (Shen et al., 2020; Deng et al., 2022) treatment to demonstrate whether *Cul3* modulates neuronal viability, morphology, or synapse formation via caspase-3 activity in neurons.

## Materials and methods

### Animals

**Figure 1:**
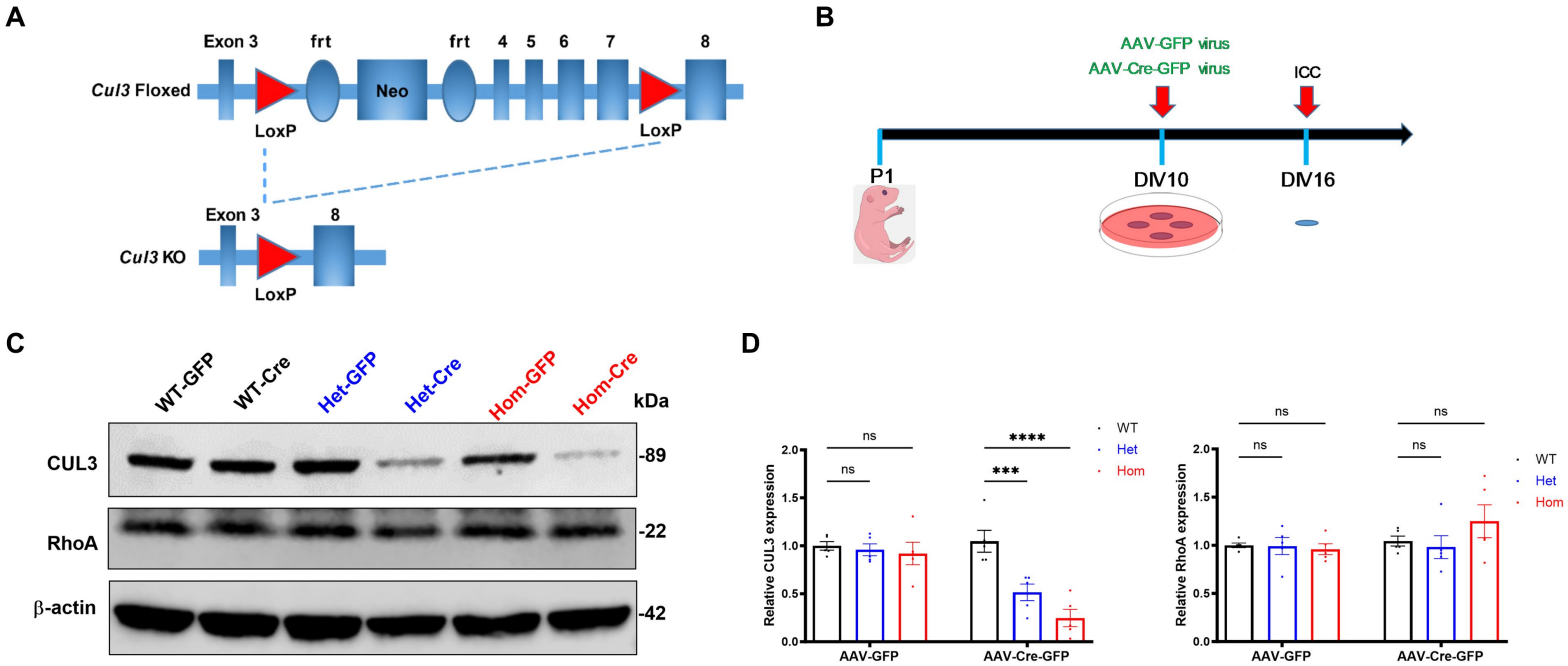
*Cul3* knockout strategy and validation in primary cultured hippocampal neurons. **(A)** Schematic representation of f*Cul3* mouse model and viral transduction Cre-LoxP knockout strategy. Exons 3–8 of *Cul3* are represented along with loxP, neomycin-resistance, and frt (flippase recognition target) sites. Transduction of neurons with AAV-Cre-GFP results in recombination and loss of *Cul3* exons 4–7. AAV-GFP transduction is used as a negative control (not depicted). **(B)** Experimental timeline of neuronal culture and AAV transduction. f*Cul3* mouse pups are sacrificed at P1, cultures transduced with AAV viruses at DIV10, then examined for outcome measures at DIV16, 6 days after transduction (ICC, immunocytochemistry). **(C,D)** Representative immunoblot [left, **(C)**] and graph of group data [right, **(D)**] for anti-CUL3,anti-β-actin and anti-RhoA blots following either AAV-Cre-GFP (WT-Cre, Het-Cre, Hom-Cre) or AAV-GFP (WT-GFP, Het-GFP, Hom-GFP) transduction in cultured f*Cul3* hippocampal neurons. *p < 0.05, **p < 0.01, ***p < 0.001, ****p < 0.0001, ordinary two-way AVONA with Dunnett’s multiple comparisons test; bars represent mean ± SEM, 5 separately derived neuronal cultures.

f*Cul3* mice (Figure 1A) were created in the Jeffrey D. Singer Lab at Portland State University (McEvoy et al., 2007) and were bred as heterozygous pairs to obtain WT, Het and Hom littermate pups. These f*Cul3* mice were originally made in a 129S1 genetic background (Jackson Labs Stock No: 002448) and then backcrossed to a C57BL/6 J background for at least 6 or more generations. All animal procedures were in accordance with the guidelines of the US National Institutes of Health Guidelines for the Care and Use of Laboratory Animals and approved by the Institutional Animal Care and Use Committee (IACUC) of UAB. Genotype of the floxed *Cul3* gene was identified by PCR using four primers as follows:

WT (F): 5’-GAACTCAGTGCATGGTTGGA-3′;
WT (R): 5’-ACACCATGGACTATGAACATGC-3′;
Mutant (F): 5’-AGCCAACGCTATGTCCTGAT-3′;
Mutant (R): 5’-TCCTGCCGAGAAAGTATCCA-3′.

WT primers produced a 382 bp band while mutant primers produced a 349 bp band.

### Antibodies and reagents

Antibodies were used at the following dilutions from the following companies: rabbit anti-vGluT1 (1:500, SYSY, #135305), mouse anti-PSD95 (1:500, Millipore, #MAB1598), guinea pig anti-vGAT (1:500, SYSY, #131005), rabbit anti-gephyrin (1:500, SYSY, #147008), goat anti-GFP (1:1000, Novus Biological, #NB100-1770), mouse anti-Cul3 (1:500, BD Biosciences, #611848), mouse anti-RhoA (1:500, Abnova, #H00000387-M08), rabbit anti-cleaved caspase-3 (1:500, Cell Signaling, #9661), rabbit anti-p-mTOR (Ser2448) (1:500, Cell Signaling, #2971), rabbit anti-mTOR (1:500, Cell Signaling, #2983), rabbit anti-GluA1 (1:500, Millipore, #AB1504), rabbit anti-GRIN2A (1:500, Millipore, #07–632), rabbit anti-GABAAR α1 (1:500, Sigma, #G4416), mouse anti-β-actin (1:1000, MP Biomedicals, #691001). Secondary antibodies for immunocytochemistry were purchased from Jackson ImmunoResearch Laboratories (488 goat anti-rabbit, #111–545–003; Cy3 donkey anti-mouse, #715–165–150; Cy3 donkey anti-guinea pig, #706–165–148; 647 donkey anti-rabbit, #711–605–152) and Secondary antibodies for Western-Blot were purchased from LI-COR (goat anti-mouse-680, #926–68070 and goat anti-rabbit-800, #926–32211). Z-DEVD-FMK was purchased from R&D Systems (#FMK004) and added in cell cultures at a final concentration of 50 μM (Bieri et al., 2018; Alharris et al., 2019) in DMSO and Neurobasal A medium (Gibco, #10888022) supplemented with 10% horse serum (Gibco, #26050–070), which was considered as vehicle. Calcein AM was purchased from Invitrogen (#C34851) and added in cell cultures at a final concentration of 5 μM (Siveen et al., 2014; Huang et al., 2019).

### AAV-virus production and transduction

AAV-Cre-GFP virus (AAV.CMV.HI.eGFP-Cre.WPRE.SV40, #105545-AAV2) and AAV-GFP virus (AAV.CMV.PI.EGFP.WPRE.bGH, #105530-AAV2) were purchased from Addgene. 1 μL virus at titer ≥7 × 10^12^ viral genomes/mL were used to transduce neurons cultured in each well of the 12-well plates at days *in vitro* (DIV) 10 followed by biochemical analyses or immunocytochemistry 6 days after transduction (Figure 1B).

### Neuron culture and immunocytochemistry

Dissociated mouse primary hippocampal neurons (containing both excitatory and inhibitory neurons without glia) were cultured as previously described (Xu et al., 2010). Briefly, mouse hippocampus was dissected in HBSS Solution (Gibco, #14175095) at postnatal day 1 (P1) and digested into single cells by 2 mg/mL Papain (Worthington, #LS003127) for 20 min with 0.5 mg/mL of DNase I (Sigma Aldrich, #DN25) added during digestion. The neurons were plated on 12 mm coverslips coated with PDL (poly-D-lysine, Millipore Sigma, #A003E). Neurobasal A medium (Gibco, #10888022) supplemented with 10% horse serum (Gibco, #26050–070) and 1% Penicillin–Streptomycin (Invitrogen, #15140–142) were added, and coverslips were then incubated in a humidified CO_2_ (5%) incubator at 37° *C.* Medium was totally changed to Neuralbasal A medium supplemented with 2% B27 (Gibco, #17504–044), 1x GlutaMax (Invitrogen, #35050–061) and 1% Penicillin–Streptomycin after 1 h, and the medium was then half-changed 2 times per week. Cytarabine (Ara-C, Millipore, #147–94–4) was added at a concentration of 0.65 μM on DIV3 to inhibit glial cell proliferation.

For morphological experiments, neurons were fixed in 4% paraformaldehyde (Electron Microscopy Sciences, #15714) and 4% sucrose in Phosphate-buffered saline (PBS) for 15 min at room temperature (RT). Neuronal membranes were permeated with 0.2% triton X-100 in PBS (pH 7.3) for 10 min at RT. Then neurons were blocked in 10% Normal Donkey Serum (NDS, Jackson ImmunoResearch Laboratories, #017–000–121) in PBS for 2 h at RT (or 4°C overnight) and incubated with primary antibody at predetermined dilution in 3% NDS in PBS at 4°C overnight in a dark chamber. Samples were then washed 3 times with PBS after the cells were incubated with secondary antibodies for 1 h at RT. After 3 washes with PBS for 10 min each, neurons were mounted in Mowiol 4–88 mounting solution containing 6 g glycerol (Fisher Scientific, #G33), 2.4 g Mowiol 4–88 (Calbiochem, #475904), 6 mL ddH_2_O, 12 mL 0.2 mol/L Tris (Fisher Scientific, #BP152), and 2.5% DABCO (1,4-diazabicyclo[2.2.2]octane, Sigma Aldrich, #27802).

### Immunoblotting

Crude lysates were collected in 2× Laemmli sample buffer (Bio-Rad, #1610737), supplemented with 2-Mercaptoethanol (Bio-Rad, #1610710), and boiled at 100°C for 5 min. Protein extracts were separated by SDS-PAGE and then transferred to a nitrocellulose membrane (Bio-Rad, #1704159). Membranes were blocked with Intercept Blocking Buffer (LI-COR, #927–60001) for 2 h at RT. Membranes were then incubated with the corresponding primary antibodies at 4°C overnight. The membranes were imaged on a LI-COR Odyssey CLx Imaging System after incubating with corresponding secondary antibodies. β-actin was used as an internal control with all protein levels normalized to the levels of these internal controls followed by normalizing all values to the average of the WT signals.

### Microscopy, image processing, and analysis

Slides were visualized under a Zeiss LSM-800 Airyscan confocal microscope. Images were captured with the same parameters and analyzed blind to treatment/genotype. Control and experimental groups were processed in parallel. Branching of GFP-positive hippocampal neuron dendrites was measured using Neurolucida 360 software (MBF Bioscience). Spines on a single randomly selected secondary dendrite from each neuron were counted, spine density and spine size were measured by Neurolucida 360 software. Spines were classified into four subtypes as previous described (Harris et al., 1992; Hering and Sheng, 2001; Rochefort and Konnerth, 2012; Xia et al., 2023): thin, stubby, mushroom and filopodia. The classifying settings of Neurolucida 360 for spine subtypes were as follows: head to neck ratio = 1.1; length to head ratio = 2.5; mushroom head size = 0.35 μm; filopodium length = 3 μm. If the ratio of the total length of the protrusion to the width of base of the protrusion was smaller than 2.5, the spine was classified as stubby; if the ratio was larger than 2.5, but the head diameter < 0.35 μm, the spine was classified as thin. A mushroom spine was classified as a spine with a neck and a head diameter > 0.35 μm. A filopodia spine was classified as a long, thin spine without a clear head and a total length smaller than 3 μm (Xia et al., 2023). Sholl analysis was based on the number of intersections per radius (10 μm) of shell. Numbers of dendrites, total dendritic length and axon length were measured using confocal microscope images including all dendrites determined via Neurolucida 360. Number of dendrites was defined as all the primary, secondary, tertiary, etc. dendrites that were traced from cell body to the neurite terminal using Neurolucida 360 software. Both excitatory and inhibitory synaptic puncta were counted on every dendrite of each neuron using the same thresholds. The percentage of PSD95 overlapping vGluT1 and of gephyrin overlapping vGAT were performed by Neurolucida 360 software for colocalization analysis.

### Statistics

Statistics were carried out using Graphpad Prism 10.2.2 (San Diego, CA, United States). Multiple comparisons were carried out using the ordinary two-way AVONA with Dunnett’s multiple comparisons test with genotype and treatment as the main variables. For Sholl analysis, spine density analysis, caspase-3 activity test and cell viability test, 30 neurons in total from 3 separately derived neuronal cultures were analyzed. For synaptic puncta density and colocalization analysis, 45 neurons in total from 3 separately derived neuronal cultures were analyzed. Blinded measurements were performed for all comparisons, see Supplementary Table S1 for detailed statistics. *p < 0.05, **p < 0.01, ***p < 0.001, ****p < 0.0001, graphs depict mean ± SEM (Standard Error of Mean).

## Results

### *Cul3* deletion from f*Cul3* mouse primary cultured hippocampal neurons

Because homozygous deletion of *Cul3* in mice leads to embryonic lethality (Singer et al., 1999), we used the Cre-loxP system and cre recombinase expression to knockout *Cul3* in hippocampal neurons cultured at P1 (Figures 1A,B). Following 10 days *in vitro* (DIV10) to establish the neurons in culture (Grabrucker et al., 2009), AAV-Cre-GFP or corresponding negative control AAV-GFP were applied to the cultures for 6 days (Figure 1B) during the period of peak synapse formation, ~DIV14 (Han and Stevens, 2009). Six days after AAV transduction (DIV16), we verified the efficiency of *Cul3* deletion at the protein level by immunoblots (Figure 1C). AAV-Cre-GFP transduction effectively reduced CUL3 expression in *Cul3* heterozygous hippocampal cultures to 52% of the AAV-Cre-GFP transduced WT control level and to 25% of the control level in *Cul3* homozygous cultures (Figure 1D). RhoA is one of the downstream targets of CUL3, which is an essential regulator of neuronal growth and migration during early brain development, hence we also validated the RhoA expression (Lin et al., 2023). Well, no significant differences in RhoA levels were observed in AAV-Cre-GFP treated groups (Figure 1D). Thus, AAV-Cre-GFP transduction efficiently reduces CUL3 in f*Cul3* heterozygous neurons and virtually eliminates it in homozygous neurons.

### *Cul3* regulates axon and dendrite elaboration or maintenance and spine density in primary cultured hippocampal neurons

The effect of *Cul3* deletion on dendritic arborization in *Drosophila* provides conflicting results (Zhu et al., 2005; Djagaeva and Doronkin, 2009), with one study revealing that dendritic elaboration is severely impaired in *Cul3* mutants in *Drosophila* mushroom body neurons (Zhu et al., 2005) and another study showing the opposite result with loss of *Cul3* causing excessive dendritic branching in *Drosophila* peripheral nervous system (Djagaeva and Doronkin, 2009). Also, previous studies of *Cul3* heterozygous deletion in mice reach differing conclusions regarding morphological changes, some showing no effect (Rapanelli et al., 2019; Dong et al., 2020; Morandell et al., 2021; Xia et al., 2023), and others showing that *Cul3* heterozygous deletion causes decreased dendritic arborization (Amar et al., 2021).

**Figure 2:**
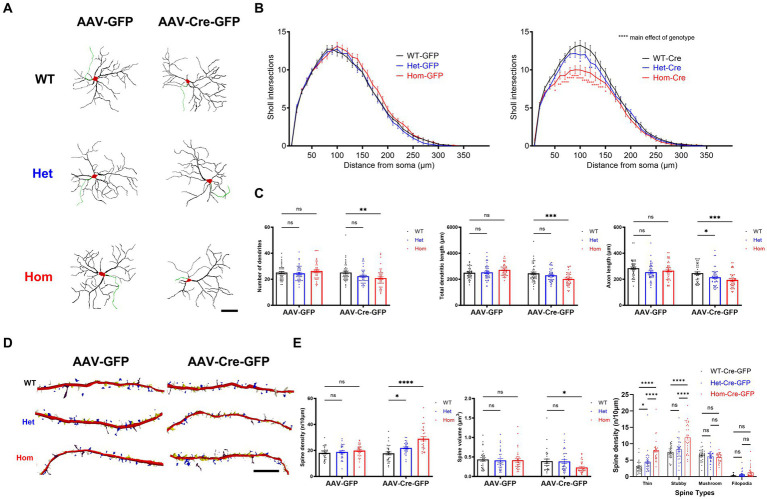
*Cul3* deletion decreases dendrite elaboration or maintenance and increases dendritic spines of reduced volume in primary cultured hippocampal neurons. **(A)** Representative tracings (derived from Neurolucida 360 analysis of confocal images) of WT, Het, or Hom f*Cul3* cultured hippocampal neurons transduced with either AAV-GFP (left column) or AAV-Cre-GFP (right column). Red, cell soma; Black, dendrite; Green, axon. Scale bar, 50 μm. **(B)** Sholl analysis of WT-Cre, Het-Cre, and Hom-Cre *Cul3* knockouts (right) demonstrates significantly decreased dendritic branching in both Het and Hom *Cul3* mutant neurons. No change in dendritic branching is observed in control AAV-GFP transduced f*Cul3* neurons (left; WT-GFP, Het-GFP, Hom-GFP). **(C)** Number of dendrites (left), total dendritic length (middle) and axon length (right) significantly decreased in *Cul3* homozygous knockout neurons (Hom-Cre) compared to control, f*Cul3* wildtype treated with AAV-Cre (WT-Cre). **(D)** Representative tracings (Neurolucida 360 analysis of confocal images) of WT, Het, and Hom f*Cul3* cultured hippocampal neuron dendritic spines transduced with AAV-GFP or AAV-Cre-GFP. White, thin; yellow, stubby; blue, mushroom; brown, filopodia. Scale bar, 20 μm. **(E)** Dendritic spine density significantly increased both in Het-Cre and Hom-Cre compared to control wildtype f*Cul3* neurons transduced with AAV-Cre (left). Average spine volume significantly reduced in homozygous *Cul3* knockout neurons (Hom-Cre) compared to control homozygous f*Cul3* neurons transduced with AAV-GFP (middle). Density of various spine subtypes increased for thin and stubby spines only (right). *p < 0.05, **p < 0.01, ***p < 0.001, ****p < 0.0001, ordinary two-way AVONA with Dunnett’s multiple comparisons test; graphs depict mean ± SEM. *N* = 30 neurons from 3 separately derived neuronal cultures (10 neurons for each group).

Because the function of *Cul3* in dendritic morphology remains unclear and because loss of the BTB adaptor protein gene *Kctd13* also alters dendritic arborization (Escamilla et al., 2017), we proceeded to elucidate the role of *Cul3* in regulating neuronal morphology. First, we examined the effect of *Cul3* deletion on dendritic arborization (Figure 2A), demonstrating that both heterozygous and homozygous loss of *Cul3* reduced dendritic complexity as measured by the number of dendrite intersections at various distances from the soma (Sholl analysis, Figure 2B, right). No such differences were observed in control AAV-GFP-transduced WT, Het, or Hom f*Cul3* mutant neurons (Figure 2B, left). Total dendritic length was also significantly reduced in homozygous *Cul3* deletion neurons (AAV-Cre-GFP treated) compared to control homozygous f*Cul3* neurons transduced with AAV-GFP (Figure 2C, middle). Significant decrease were also observed in the number of dendrites (Figure 2C, left). Axon length was significantly reduced in homozygous *Cul3* deletion neurons (Figure 2C, right). Because mTOR (mammalian target of rapamycin) is involved in dendritic morphology (Kumar et al., 2005), we also performed immunoblots against p-mTOR and mTOR but interestingly found no p-mTOR/mTOR ratio differences among the AAV-Cre-treated groups (WT/Het/Hom, Supplementary Figure S1). These data suggest that *Cul3* modulates dendrite arborization and length as well as axonal length or maintenance.

The nature or presence of spine morphological changes after *Cul3* heterozygous deletion in mice is also inconsistent among published studies. Decreased basal spine density (decreased thin spine density) in prefrontal cortical neurons was found in one study (Rapanelli et al., 2019); another study reports increased apical spine density and unchanged basal spine density in hippocampus CA1 pyramidal neurons (Dong et al., 2020), and other studies find unchanged spine density in different brain regions (Morandell et al., 2021; Xia et al., 2023). To determine effects on early spine development, we examined effects of *Cul3* deletion on dendritic spines in cultured hippocampal neurons (Figure 2D). In contrast to the effects of *Kctd13* deletion on dendritic spines (Escamilla et al., 2017), heterozygous and homozygous deletion of *Cul3* by AAV-Cre-GFP in hippocampal neurons led to increased dendritic spine density (Figure 2E, left) but decreased average spine volume (Figure 2E, middle). We next examined which spine morphological subtypes (thin, stubby, mushroom, or filopodia) might be responsible for the increased spine density. We identified an increase in thin and stubby spines in *Cul3* Het and Hom mutant neurons, while mushroom and filopodia spine numbers are comparable among all the groups (Figure 2E, right). These findings suggest that loss of *Cul3* leads to increased formation or maintenance of dendritic spines that are on average smaller and possibly less mature, indicating a role for *Cul3* in modulation of spine density and size.

### Z-DEVD-FMK blocks *Cul3* deletion-mediated caspase-3 activity in primary cultured hippocampal neurons

Previous studies revealed that *Cul3* loss leads to a significant increase in caspase-3 activation which is thought to indicate subsequent neuronal cell death (Kaplan et al., 2010; Amar et al., 2021; Morandell et al., 2021). Previous studies also indicate that different caspase-3 activation level is a key regulatory mechanism of not only programmed cell death but also cell morphology (D'Amelio et al., 2010, 2012; Saric et al., 2022). Non-apoptotic caspase-3 functions like dendrite pruning or spine maturation also suggest that caspase-3 is a key regulatory molecule in neurogenesis and synapse formation/homeostasis (D'Amelio et al., 2010). In turn, we hypothesized that this pathway may also be a promising mechanism for altered neuronal morphology. So, we used Z-DEVD-FMK (Bieri et al., 2018; Alharris et al., 2019), a caspase-3 inhibitor peptide, to examine whether the *Cul3* deletion phenotypes can be reversed by caspase-3 inhibition.

**Figure 3:**
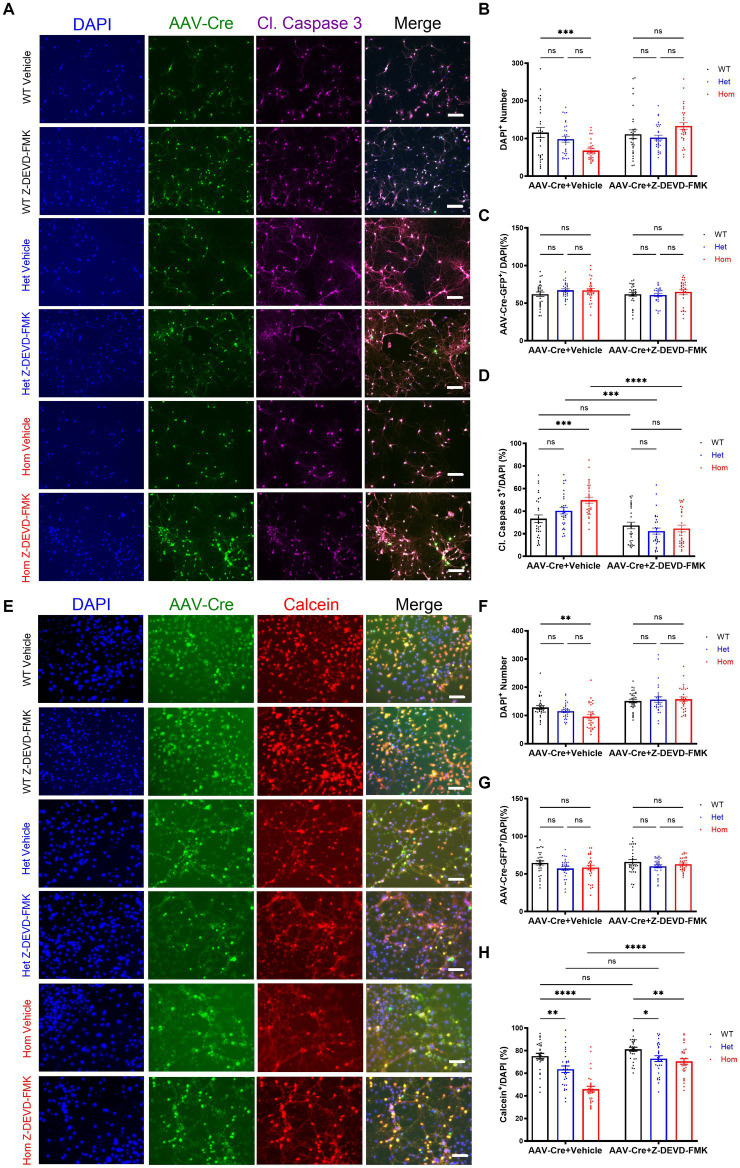
*Cul3* modulates cell viability through caspase-3 activity in primary cultured hippocampal neurons. **(A)** Representative images of WT, heterozygous, and homozygous f*Cul3* cultured hippocampal neurons treated with Z-DEVD-FMK or vehicle transduced with AAV-Cre-GFP viruses (WT-Cre, Het-Cre, Hom-Cre). DAPI, cell nucleus marker. Cl. Caspase-3, cleaved caspase-3. Scale bar, 50 μm. **(B)** Neuron number counted based on DAPI signal. **(C)** AAV virus transduction efficiency was comparable among all groups. **(D)** Caspase-3 positive signal was divided by DAPI signal to determine the caspase-3-positive cell rate. Increased caspase-3 activity is largely reversed by Z-DEVD-FMK treatment in Hom group. **(E)** Representative images of WT, heterozygous, and homozygous f*Cul3* cultured hippocampal neurons treated with Z-DEVD-FMK or vehicle transduced with AAV-Cre-GFP viruses (WT-Cre, Het-Cre, Hom-Cre). Calcein, viable cell marker. Scale bar, 50 μm. **(F)** Neuron number based on DAPI signal. **(G)** AAV virus transduction efficiency was comparable among all groups. **(H)** Calcein positive signal was divided by DAPI signal to determine viable cell rate. Decreased viable cell rate is largely reversed by Z-DEVD-FMK treatment in both Het and Hom group. *p < 0.05, **p < 0.01, ***p < 0.001, ****p < 0.0001, ordinary two-way AVONA with Dunnett’s multiple comparisons test; graphs depict mean ± SEM. *N* = 30 neurons from 3 separately derived neuronal cultures (10 neurons for each group).

First, we examined caspase-3 activation in our *Cul3* depleted neurons via immunohistochemistry and normalized caspase-3-activated neurons to all DAPI-stained neurons (Figure 3A). We validated the cell number based on DAPI-stained neurons and discovered significantly decreased cell numbers in *Cul3* homozygous deletion group. This decreased cell density was reversed by caspase-3 activity inhibition (Figure 3B), and the transduction efficiencies were comparable among all the groups (Figure 3C). We confirmed that caspase-3 activation significantly increased in the *Cul3* homozygous deletion group (Figure 3B), which was consistent with previous studies (Kaplan et al., 2010; Amar et al., 2021; Morandell et al., 2021). As a control, we also demonstrated that inhibiting caspase-3 prevented its activation (Figure 3D).

### *Cul3* modulates cell viability through caspase-3 activity in primary cultured hippocampal neurons

Because caspase-3 activation can occur without neuronal death (Acarin et al., 2007; Erturk et al., 2014; Khatri et al., 2018; Saric et al., 2022), we examined whether the number of viable neurons were changed with *Cul3* deletion. First, we confirmed that DAPI-stained cells were decreased in *Cul3* deletion cultures (Figures 3E,F), and that this decrease was reversed by caspase-3 inhibition (Figures 3E,F). Transduction efficiency was again comparable (Figure 3G). Using a viable cell marker, Calcein AM (AM: Acetoxymethyl ester) normalized to DAPI-stained cells, we identified a decrease in viable neurons in the *Cul3* Het and Hom deletion cultures that was also reversed by caspase-3 inhibition (Figure 3H). These data support the hypothesis that *Cul3* modulates neuronal viability through caspase-3 activity in neurons. In addition, we observed a qualitative appearance of increased cell aggregation in the absence of *Cul3* (Figures 3A,E), revealing potential mechanisms of cytoskeleton dysregulation in the absence of *Cul3* in primary neuronal cultures, which are consistent with the previous findings (Amar et al., 2021; Morandell et al., 2021).

### *Cul3* regulates dendritic complexity & spines via caspase-3

**Figure 4:**
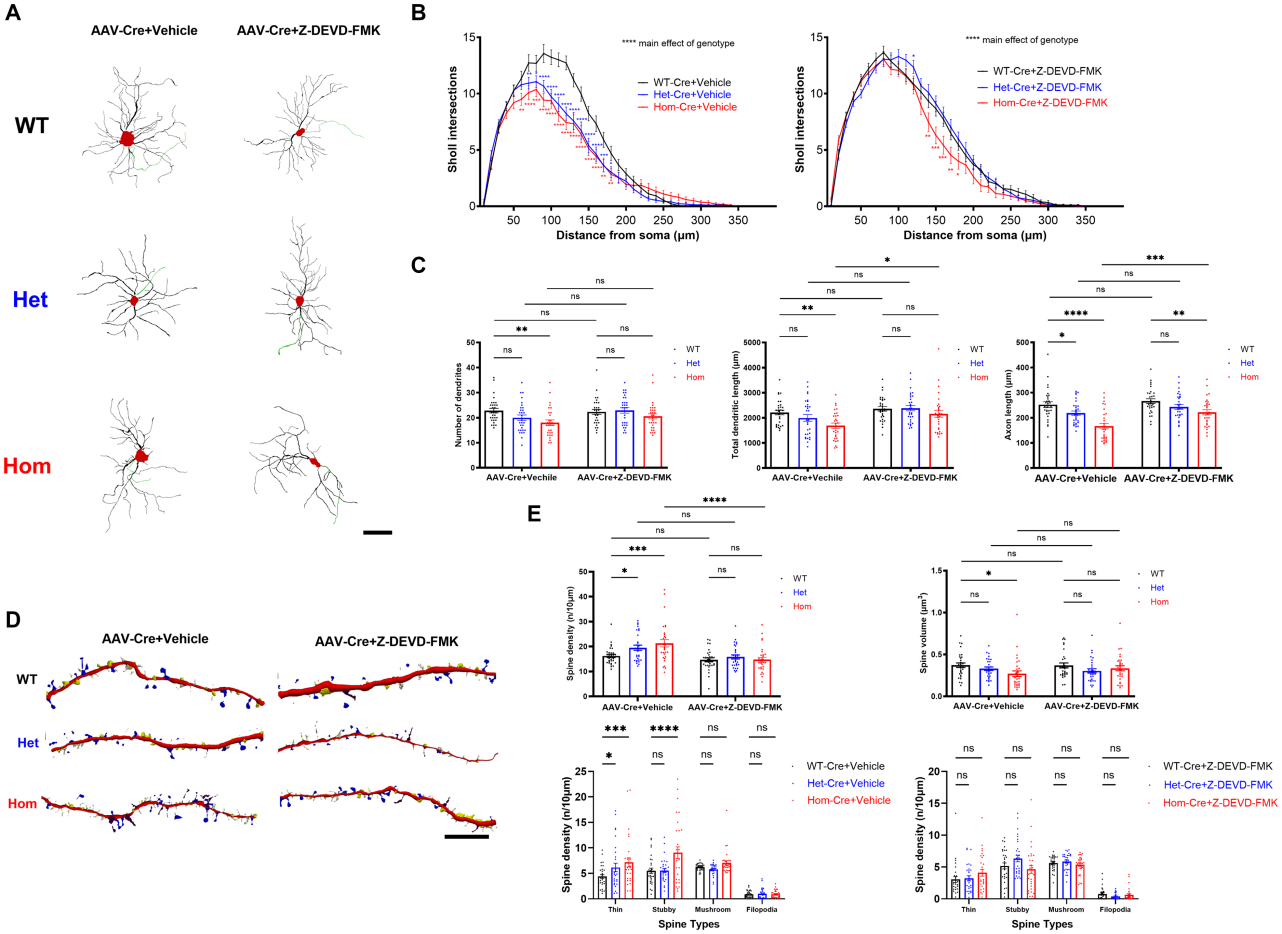
*Cul3* regulates dendritic complexity and spine generation through caspase-3 activity in primary cultured hippocampal neurons. **(A)** Representative tracings (derived from Neurolucida 360 analysis of confocal images) of WT, heterozygous, and homozygous f*Cul3* cultured hippocampal neurons treated with Z-DEVD-FMK or vehicle transduced with AAV-Cre-GFP viruses (WT-Cre, Het-Cre, Hom-Cre). Red, cell soma; Black, dendrite; Green, axon. Scale bar, 50 μm. **(B)** Sholl analysis of WT-Cre, Het-Cre, and Hom-Cre *Cul3* knockouts (left) demonstrates significantly decreased dendritic branching in both Het and Hom *Cul3* mutant neurons. Decreased dendritic branching is largely reversed by Z-DEVD-FMK treatment in both Het and Hom group (right). **(C)** Number of dendrites (left), total dendritic length (middle) and axon length (right) significantly decreased in *Cul3* homozygous knockout neurons (Hom-Cre) compared to control, f*Cul3* wildtype treated with AAV-Cre (WT-Cre). Decreased number of dendrites, total dendritic length and axon length were largely reversed by Z-DEVD-FMK treatment in Hom group. **(D)** Representative tracings (Neurolucida 360 analysis of confocal images) of WT, Het, and Hom f*Cul3* cultured hippocampal neuronal dendritic spines treated with Z-DEVD-FMK or vehicle transduced with AAV-Cre-GFP viruses (WT-Cre, Het-Cre, Hom-Cre). White, thin; yellow, stubby; blue, mushroom; brown, filopodia. Scale bar, 20 μm. **(E)** Dendritic spine density significantly increased in heterozygous and homozygous *Cul3* knockout neurons were reversed by Z-DEVD-FMK treatment (upper left). Average spine volume significantly reduced in homozygous *Cul3* knockout neurons was reversed by Z-DEVD-FMK treatment (upper right). Increased spine density with Cul3 deletion was largely due to increased thin and stubby spines increasing (lower left), and this was reversed by Z-DEVD-FMK (lower right). *p < 0.05, **p < 0.01, ***p < 0.001, ****p < 0.0001, ordinary two-way AVONA with Dunnett’s multiple comparisons test; graphs depict mean ± SEM. *N* = 30 neurons from 3 separately derived neuronal cultures (10 neurons for each group).

Previous studies revealed that caspase-3 activity regulates not only cell death, but also cell morphology (Kaplan et al., 2010; Erturk et al., 2014; Guo et al., 2016; Hollville and Deshmukh, 2018; Amar et al., 2021; Morandell et al., 2021). Thus, we hypothesized that some of our neuronal morphology changes may result from elevated caspase-3 activity as well (Saric et al., 2022). To test this, we inhibited caspase-3 and examined neuronal morphology. Replicating our previous findings, Hom *Cul3* mutant neurons demonstrated reduced dendrite and axon length as well as reduced dendrite complexity (Figures 4A–C), both Het and Hom *Cul3* mutation increased spine density (Figures 4D,E), and Hom *Cul3* mutant neurons demonstrated decreased spine volume (Figures 4D,E). The increased spine density mainly consists of thin and stubby spines (Figures 4D,E). Consistent with our hypothesis, caspase-3 inhibition largely reversed both dendrite, axon (Figures 4A–C) and spine (Figures 4D,E) morphological changes. These data indicate that *Cul3* regulates dendritic complexity and spine generation at least in part via modulation of caspase-3 activity.

### *Cul3* deletion does not impact excitatory synaptic puncta formation in primary cultured hippocampal neurons

**Figure 5:**
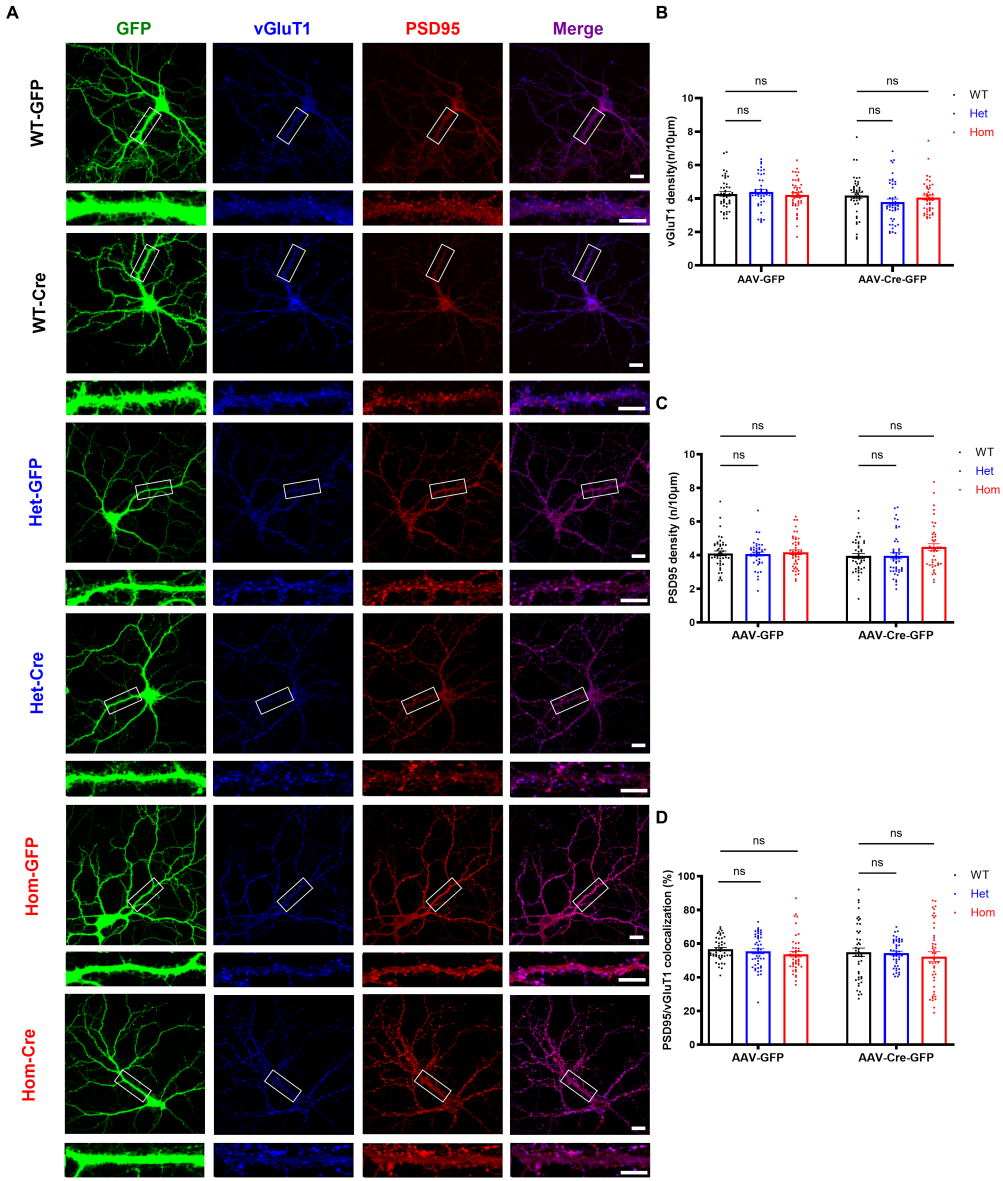
*Cul3* homozygous deletion and heterozygous reduction lead to unchanged excitatory synapses in primary cultured hippocampal neurons. **(A)** Representative images of excitatory synaptic puncta in WT, heterozygous, and homozygous f*Cul3* cultured hippocampal neurons transduced with either AAV-GFP (WT-GFP, Het-GFP, Hom-GFP) or AAV-Cre-GFP (WT-Cre, Het-Cre, Hom-Cre). Scale bars represent 50 μm for the larger images and 20 μm for the magnified dendrite images. **(B)** Presynaptic excitatory synapse marker vGluT1 puncta density is comparable in both heterozygous and homozygous *Cul3* knockout neurons (Het-Cre and Hom-Cre) compared to control heterozygous and homozygous f*Cul3* neurons transduced with AAV-GFP (Het-GFP and Hom-GFP). **(C)** Postsynaptic excitatory synapse marker PSD95 puncta density is unchanged in both heterozygous and homozygous *Cul3* knockout neurons (Het-Cre and Hom-Cre) compared to control heterozygous and homozygous f*Cul3* neurons transduced with AAV-GFP. **(D)** Percentage of co-localized PSD95/vGluT1 puncta is unchanged in homozygous *Cul3* knockout neurons (Hom-Cre) compared to homozygous f*Cul3* neurons transduced with AAV-GFP (Hom-GFP). *p < 0.05, **p < 0.01, ***p < 0.001, ****p < 0.0001, ordinary two-way AVONA with Dunnett’s multiple comparisons test; graphs depict mean ± SEM. *N* = 45 neurons from 3 separately derived neuronal cultures (15 neurons for each group).

Because the combination of decreased total dendritic length and complexity, increased density of spines, and decreased volume of spines was difficult to interpret in terms of synapse number, we next examined whether *Cul3* deletion impairs excitatory synaptic puncta formation. Thus, we labeled pre- and post-synaptic excitatory markers (vGluT1 and PSD95) (Figure 5A). Both vGulT1 (Figure 5B) and PSD95 (Figure 5C) puncta density was unchanged in both heterozygous and homozygous *Cul3* deletion hippocampal neurons compared to heterozygous and homozygous f*Cul3* AAV-GFP transduced control neurons (Figures 5B,C). The percentage of co-localized PSD95/vGluT1 synaptic puncta compared to isolated pre- or post-synaptic puncta were comparable in homozygous *Cul3* deletion neurons as well (Figure 5D). Because a previous report suggested decreased NMDAR-EPSC (excitatory postsynaptic currents) and unchanged AMPAR-EPSC input/output curves with a conditional heterozygous *Cul3* deletion (Rapanelli et al., 2019), but we found comparable NMDA/AMPA current ratio between WT and *Cul3* heterozygous neurons (Xia et al., 2023), so we performed immunoblots against NMDAR subunit GRIN2A and AMPAR subunit GluA1 and found no differences among the AAV-Cre-treated groups (WT/Het/Hom, Supplementary Figures S1A,C,D). These data suggest that *Cul3* deletion does not significantly influence excitatory synaptic puncta density or colocalization in developing hippocampal neurons.

### *Cul3* deletion impairs inhibitory synaptic puncta formation or maintenance

**Figure 6:**
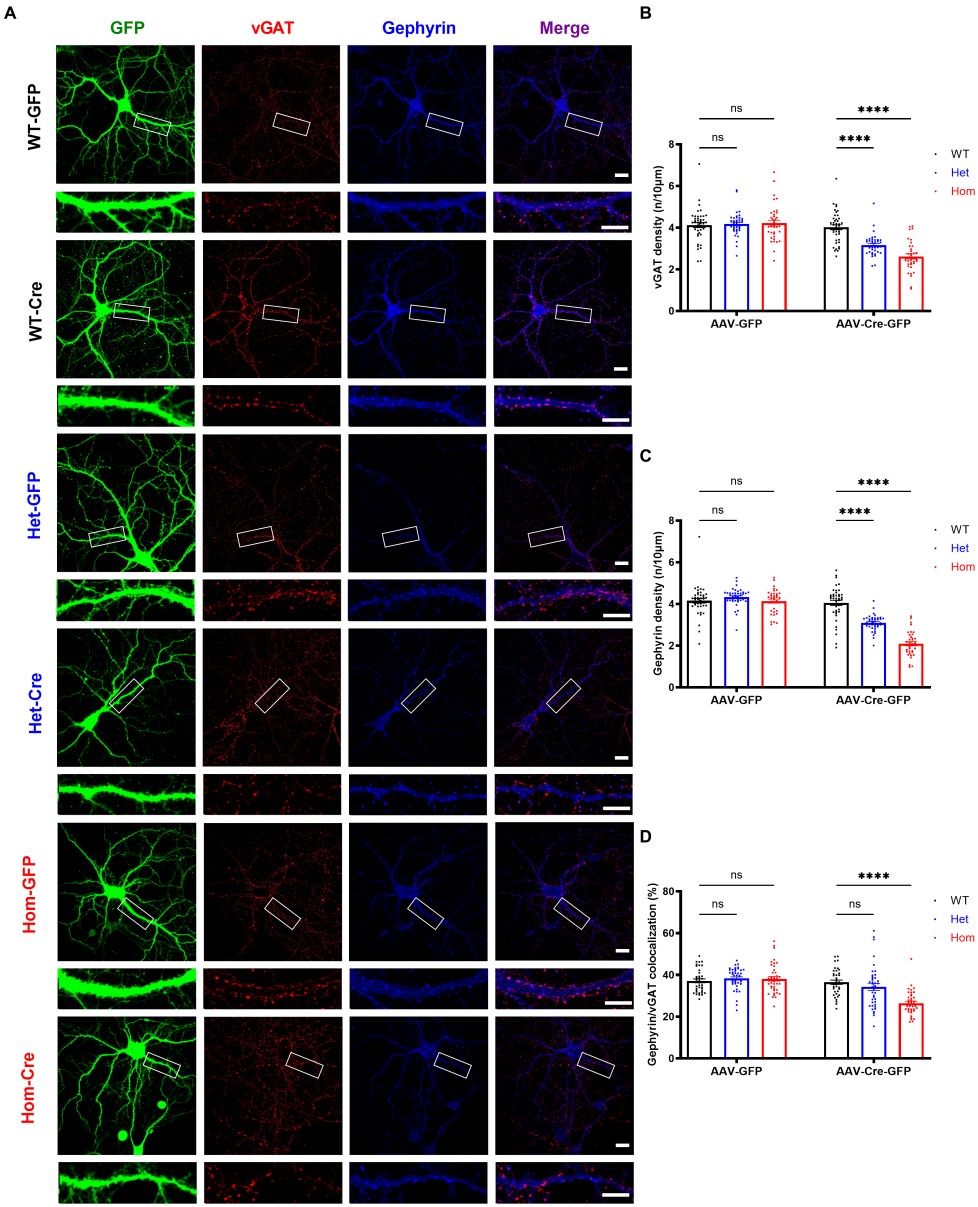
*Cul3* homozygous deletion and heterozygous reduction lead to reduced inhibitory synapses in primary cultured hippocampal neurons. **(A)** Representative images of inhibitory synaptic puncta in WT, heterozygous, and homozygous f*Cul3* cultured hippocampal neurons transduced with either AAV-GFP (WT-GFP, Het-GFP, Hom-GFP) or AAV-Cre-GFP (WT-Cre, Het-Cre, Hom-Cre). Scale bars represent 50 μm for the larger images and 20 μm for the magnified dendrite images. **(B)** Presynaptic inhibitory synapse marker vGAT puncta density significantly decreased in both heterozygous and homozygous *Cul3* knockout neurons (Het-Cre and Hom-Cre) compared to control heterozygous and homozygous f*Cul3* neurons transduced with AAV-GFP (Het-GFP and Hom-GFP). **(C)** Postsynaptic inhibitory synapse marker gephyrin puncta density significantly decreased in both heterozygous and homozygous *Cul3* knockout neurons (Het-Cre and Hom-Cre) compared to control heterozygous and homozygous f*Cul3* neurons transduced with AAV-GFP (Het-GFP and Hom-GFP). **(D)** Percentage of co-localized gephyrin/vGAT puncta significantly decreased in homozygous *Cul3* knockout neurons (Hom-Cre) compared to homozygous f*Cul3* neurons transduced with AAV-GFP (Hom-GFP). *p < 0.05, **p < 0.01, ***p < 0.001, ****p < 0.0001, ordinary two-way AVONA with Dunnett’s multiple comparisons test; graphs depict mean ± SEM. *N* = 45 neurons from 3 separately derived neuronal cultures (15 neurons for each group).

We next examined the density of inhibitory pre- and post-synaptic markers (vGAT and gephyrin) in *Cul3* deletion hippocampal neurons (Figure 6A). In contrast to excitatory synaptic puncta, both inhibitory pre-synaptic marker puncta (Figure 6B) and inhibitory post-synaptic marker puncta (Figure 6C) were significantly reduced in both heterozygous and homozygous *Cul3* deletion neurons (Figures 6B,C). The percentage of co-localized gephyrin/vGAT inhibitory synaptic puncta compared to isolated pre- or post-synaptic puncta was significantly reduced only in homozygous *Cul3* deletion neurons (Figure 6D). We also measured GABAAR α1 subunits and found a significant reduction in protein expression with both Het and Hom *Cul3* reduction/deletion (Supplementary Figures S1A,F). Taken together, these results suggest that *Cul3* regulates the formation or maintenance of inhibitory synaptic puncta in early development of hippocampal neurons.

### *Cul3* regulates inhibitory synaptic puncta formation or maintenance via caspase-3

**Figure 7:**
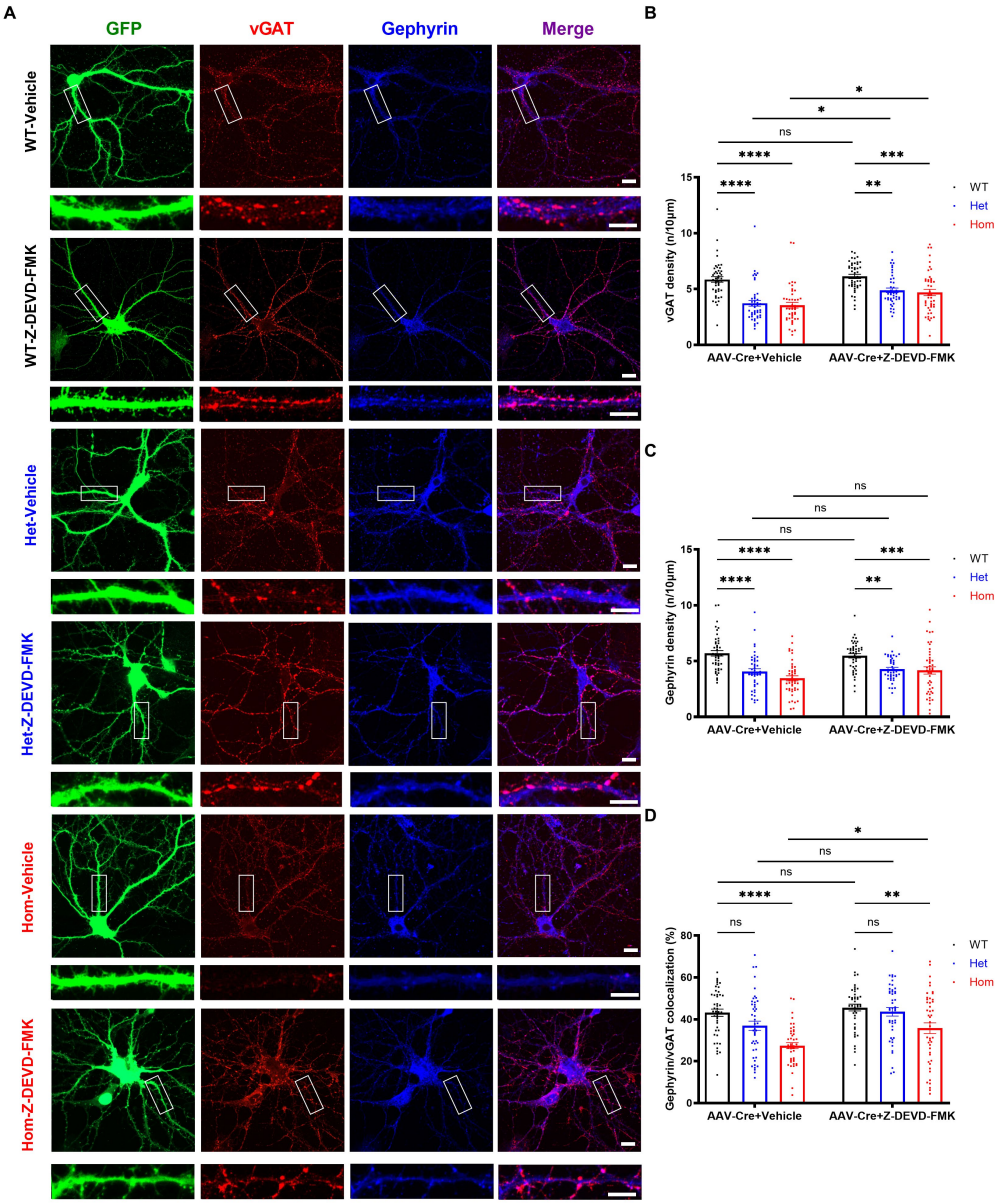
Caspase-3 inhibition partially rescues *Cul3* deletion mediated reduced inhibitory synapses in primary cultured hippocampal neurons. **(A)** Representative images of inhibitory synaptic puncta in WT, heterozygous, and homozygous f*Cul3* cultured hippocampal neurons transduced with either Z-DEVD-FMK or vehicle transduced with AAV-Cre-GFP viruses (WT-Cre, Het-Cre, Hom-Cre). Scale bars represent 50 μm for the larger images and 20 μm for the magnified dendrite images. **(B)** Presynaptic inhibitory synapse marker vGAT puncta density significantly decreased in both heterozygous and homozygous *Cul3* knockout neurons (Het-Cre and Hom-Cre) compared to control f*Cul3* neurons (WT-Cre). Decreased vGAT density is largely reversed by Z-DEVD-FMK treatment in both Het and Hom group. **(C)** Postsynaptic inhibitory synapse marker gephyrin puncta density significantly decreased in both heterozygous and homozygous *Cul3* knockout neurons (Het-Cre and Hom-Cre) compared to control f*Cul3* neurons (WT-Cre). Decreased gephyrin density is largely reversed by Z-DEVD-FMK treatment in both Het and Hom group. **(D)** Percentage of co-localized gephyrin/vGAT puncta significantly decreased in homozygous *Cul3* knockout neurons (Hom-Cre) which can be partially rescued by Z-DEVD-FMK. *p < 0.05, **p < 0.01, ***p < 0.001, ****p < 0.0001, ordinary two-way AVONA with Dunnett’s multiple comparisons test; graphs depict mean ± SEM. *N* = 45 neurons from 3 separately derived neuronal cultures (15 neurons for each group).

In addition to regulation of cell death and cell morphology, caspase-3 is also believed to modulate synaptic plasticity (Li et al., 2010; Wang et al., 2014; Gu et al., 2021; Fieblinger et al., 2022) and possibly inhibitory synapse elimination (Miller-Fleming et al., 2016). Thus, we hypothesized that the changes in inhibitory synaptic puncta number may result from elevated caspase-3 activity. To test this, we inhibited caspase-3 and examined inhibitory synaptic puncta. Replicating our previous findings, both Het and Hom *Cul3* mutant vehicle-treated neurons demonstrated reduced pre- and post-synaptic inhibitory puncta density, and Hom *Cul3* mutant vehicle-treated neurons revealed decreased percentage of co-localized gephyrin/vGAT synaptic puncta (Figures 7A–D). Interestingly, caspase-3 inhibition partially reversed both vGAT (Figures 7A,B) and gephyrin (Figures 7A,C) puncta density in both Het and Hom groups; caspase-3 inhibition also largely reversed the reduced gephyrin/vGAT inhibitory synaptic puncta co-localization in Hom *Cul3* deletion neurons (Figure 7D). These findings are consistent with our hypothesis that *Cul3* participates in regulating inhibitory synaptic puncta formation or maintenance in part via modulation of caspase-3 activity.

## Discussion

Overall, our results indicate a role for *Cul3* in modulation of dendritic length/complexity, axonal length, and dendritic spine and inhibitory synaptic puncta formation/maintenance in early mammalian hippocampal neuronal development. We have used developing neuronal cultures *in vitro* from an f*Cul3* model paired with AAV-Cre transduction due to the embryonic lethal nature of constitutive *Cul3* homozygous deletion (Singer et al., 1999). For genes such as *Cul3* for which neuronal function remains underexplored, homozygous deletion is among the best tools to elucidate function, particularly for autism risk genes (Blundell et al., 2009; Etherton et al., 2009; Zhou et al., 2009; Blundell et al., 2010; Espinosa et al., 2015; Escamilla et al., 2017; Jaramillo et al., 2017). Thus, our approach adds to the existing literature demonstrating effects of homozygous *Cul3* deletion on neuronal function (Rapanelli et al., 2019; Dong et al., 2020; Fischer et al., 2020; Amar et al., 2021; Morandell et al., 2021).

Recent publications have examined *Cul3* genetic mouse models (Rapanelli et al., 2019; Dong et al., 2020; Amar et al., 2021; Morandell et al., 2021), largely in the heterozygous state, and *Cul3* neurons derived from human inducible pluripotent stem cells (Fischer et al., 2020). Contrary to our results in developing hippocampal neuronal cultures, a recent study on Emx1-Cre/f*Cul3* heterozygous mice showed that there was no change in number of intersections, number of dendrites and average dendritic length of prefrontal cortical pyramidal neurons much later in development *in vivo* (Rapanelli et al., 2019). Similarly, another study in pyramidal neurons of adult *Cul3^+/−^* mice somatosensory cortex also revealed normal dendrite morphology (Morandell et al., 2021). An *in vitro* study found a significant decrease of total dendrite length and number of dendrites in the *Cul3^+/−^* mice DIV14 cultured primary cortical neurons (Amar et al., 2021). None of these studies are directly comparable to ours due to the use of different brain regions and conditions.

Several lines of evidence in this study support a role for *Cul3* in dendrite regulation in hippocampal neurons in culture. Homozygous loss of *Cul3* leads to a decrease in total dendritic length and decreased dendritic branching by Sholl analysis. At the same time, synaptic spine density is increased, but spine volume is decreased in our cultured hippocampal neurons, and we have demonstrated that this spine density increase is largely mediated via immature appearing spines. Decreased dendritic length is expected to decrease dendritic area, potentially leading to decreased area for synaptic connections, yet spine density is increased, perhaps as a homeostatic compensation for decreased dendrites. It is possible that we are seeing the emergence of immature appearing spines that will later mature and make up for the loss of dendritic complexity. Actinfilin (a CUL3 substrate adaptor) is critical for enlargement and maintenance of dendritic spines and controls both the synaptic localization and the size of synaptic GluR6-containing kainate receptor clusters in hippocampal neurons, and actinfilin-CUL3-mediated degradation is important for regulating neuronal GluR6 surface expression (Salinas et al., 2006), which is strengthening the importance of *Cul3*’s role in actin cytoskeleton dynamics and a promising future direction for studying *Cul3*’s regulatory mechanism in cell morphology.

Our results demonstrate an increase in hippocampal neuron spine density in both heterozygous and homozygous *Cul3* deletion; other studies focused mainly on heterozygous *Cul3* deletion with varying results (Jaramillo et al., 2017; Rapanelli et al., 2019; Dong et al., 2020; Amar et al., 2021). The study on Emx1-Cre/f*Cul3* heterozygous mice showed a significant decrease in spine density of prefrontal cortical pyramidal neurons (Rapanelli et al., 2019). Another study on GFAP-Cre f*Cul3* heterozygous mice indicated significant increases in apical spine density and no change in basal spine density of hippocampal CA1 pyramidal neurons (Dong et al., 2020). A study in pyramidal neurons of adult *Cul3^+/−^* mice somatosensory cortex revealed unchanged spine density (Morandell et al., 2021). Again, it is difficult to compare these studies performed under different conditions and at different timepoints in development.

Previous studies revealed that *Cul3* loss leads to a significant increase of caspase-3 activation (Kaplan et al., 2010; Amar et al., 2021; Morandell et al., 2021) and caspase-3 activation level is a key regulatory mechanism of cell morphology and programmed cell death (Erturk et al., 2014; Hollville and Deshmukh, 2018; Saric et al., 2022). By inhibiting caspase-3 activity, we discovered that the alteration in both dendrites and spines can be largely reversed to WT control levels.

On one hand, apoptotic stimuli such as DNA damage will lead to the release of cytochrome C (cyto-C) from mitochondria into the cytosol, combine with apoptotic protease activating factor-1 (Apaf-1) and then result in the activation of caspase-9; active caspase-9 further activates downstream effector caspases like caspase-3, caspase-6 and caspase-7 to initiate apoptosis (Wang and Lenardo, 2000; Schleich and Lavrik, 2013). On the other hand, death-receptor-induced caspase-8 activation can also activate downstream effector caspases and initiate apoptosis (Wang and Lenardo, 2000; Schleich and Lavrik, 2013). A previous study revealed that death receptor ligation induces polyubiquitination of caspase-8, through interaction of the death-inducing signaling complex (DISC) with a *CUL3*-based E3 ligase (Jin et al., 2009). The activation of caspase-3 leads to plasma membrane blebbing, chromatin condensation, DNA cleavage and phosphatidylserine exposure; it then produces the morphological and biochemical characteristics of apoptotic cells (Jiang et al., 2020). Our results indicates that *Cul3* homozygous deletion significantly increases caspase-3 activity and subsequently increases cell apoptosis, which is agreeing with previous *Cul3* studies (Amar et al., 2021; Morandell et al., 2021). Caspase-3 is also thought to mediate dendrite elaboration and dendritic complexity based on previous findings (Kaplan et al., 2010; Erturk et al., 2014; Guo et al., 2016; Hollville and Deshmukh, 2018; Amar et al., 2021; Morandell et al., 2021). Previous studies also demonstrated that inhibition of caspase-3 blocks spine shrinkage and leads to enlargement of spines (D'Amelio et al., 2011; Erturk et al., 2014). In agreement with these previous findings, our data indicate that *Cul3* regulates dendritic complexity and spine generation or maintenance via caspase-3 activity.

It is becoming increasingly clear that caspase-3 regulates synaptic plasticity and contributes to learning and memory as a non-apoptotic function (Li et al., 2010; Wang et al., 2014; Gu et al., 2021; Fieblinger et al., 2022). Staining for excitatory pre- and post-synaptic markers demonstrates unchanged excitatory synaptic puncta density and co-localization in our *Cul3* homozygous deletion cultured neurons. A previous study also indicated that UNC-8 (UNC, un-coordinated) triggers a caspase-dependent mechanism to drive GABA neuron synapse elimination (Miller-Fleming et al., 2016). It is interesting that a significant decrease in inhibitory synaptic puncta occurs in heterozygous *Cul3* deletion neurons; heterozygous *Cul3* loss best mimics the genotype of ASD patients with *Cul3* loss-of-function mutations (Kong et al., 2012; O'Roak et al., 2012a,b; De Rubeis et al., 2014; Iossifov et al., 2014; Deciphering Developmental Disorders S, 2015; Sanders et al., 2015; da Silva Montenegro et al., 2020; Nakashima et al., 2020). Similarly, homozygous loss of *Cul3* also leads to a significantly decreased fraction of inhibitory pre/post-synaptic marker co-localization. Interestingly, we demonstrate that caspase-3 inhibition partially rescues the decreased inhibitory synaptic puncta in heterozygous and homozygous *Cul3* deletion neurons, revealing a potential role for *Cul3* in regulating inhibitory synapses via caspase-3 activity.

Overall, our findings demonstrate that *Cul3* regulates axon and dendrite elaboration or maintenance, neuronal viability, spine density and inhibitory synaptic puncta density via caspase-3 activity in primary cultured hippocampal neurons, furthermore, these alterations in early neuronal development can largely be rescued by caspase-3 inhibition. In conclusion, *Cul3* regulates the formation or maintenance of cell morphology, GABAergic synaptic puncta, and neuronal viability in developing hippocampal neurons in culture.

## Data availability statement

The dataset analyzed in this paper is available at the GIN repository (https://doi.org/10.12751/g-node.qdqcp3).

## Ethics statement

The animal study was approved by Institutional Animal Care and Use Committee (IACUC) of University of Alabama at Birmingham. The study was conducted in accordance with the local legislation and institutional requirements.

## Author contributions

Q-qX: Conceptualization, Data curation, Formal analysis, Funding acquisition, Investigation, Methodology, Project administration, Resources, Software, Validation, Visualization, Writing – original draft, Writing – review & editing. AS: Data curation, Formal analysis, Investigation, Methodology, Validation, Visualization, Writing – review & editing. JW: Data curation, Formal analysis, Investigation, Methodology, Validation, Visualization, Writing – review & editing. ZX: Methodology, Resources, Validation, Writing – review & editing. JS: Resources, Writing – review & editing, Methodology. CP: Conceptualization, Funding acquisition, Methodology, Project administration, Resources, Supervision, Writing – review & editing.

## Supplementary material

SUPPLEMENTARY FIGURE S1 *Cul3* homozygous deletion and heterozygous reduction decrease GABAA receptor but not ionotropic glutamate receptor expression in primary cultured hippocampal neurons. **(A–F)** Representative immunoblots **(A)** and graph of group data **(B–F)** for anti-p-mTOR, anti-mTOR, anti-GRIN2A, anti-GluA1, anti-CUL3, anti-GABAAR α1 and anti-β-actin blots following AAV-Cre-GFP (WT-Cre, Het-Cre, Hom-Cre) transduction in cultured f*Cul3* hippocampal neurons. * *p* < 0.05, ** *p* < 0.01, *** *p* < 0.001, **** *p* < 0.0001, ordinary one-way AVONA with Dunnett’s multiple comparisons test; bars represent mean ± SEM, 4 separately derived neuronal cultures.

SUPPLEMENTARY TABLE S1 Detailed statistical analysis for all comparisons.
