# Supplementary material for: Autism risk gene Cul3 alters neuronal morphology via caspase-3 activity in mouse hippocampal neurons
Source: Front Cell Neurosci. 2024 May 9;18:1320784. doi: 10.3389/fncel.2024.1320784 (PMC11129687; doi:10.3389/fncel.2024.1320784)
Supplement: SUPPLEMENTARY FIGURE S1 — Cul3 homozygous deletion and heterozygous reduction decrease GABAA receptor but not ionotropic glutamate receptor expression in primary cultured hippocampal neurons. (A–F) Representative immunoblots (A) and graph of group data (B–F) for anti-p-mTOR, anti-mTOR, anti-GRIN2A, anti-GluA1, anti-CUL3, anti-GABAAR α1 and anti-β-actin blots following AAV-Cre-GFP (WT-Cre, Het-Cre, Hom-Cre) transduction in cultured fCul3 hippocampal neurons. * p < 0.05, ** p < 0.01, *** p < 0.001, **** p < 0.0001, ordinary one-way AVONA with Dunnett’s multiple comparisons test; bars represent mean ± SEM, 4 separately derived neuronal cultures. [file Data_Sheet_1.pdf]

Supplementary material

Autism Risk Gene *Cul3* Alters Neuronal Morphology via Caspase-3 Activity in Mouse Hippocampal Neurons

Qiang-qiang Xia[1], Anju Singh[1], Jing Wang[1], Zhong Xin Xuan[1], Jeffrey D. Singer[2], Craig M. Powell[1*]

[1]Department of Neurobiology, Marnix E. Heersink School of Medicine & Civitan International Research Center, University of Alabama at Birmingham, Birmingham, AL, United States.

[2]Department of Biology, Portland State University, Portland, OR, United States.

*Corresponding author: Craig M. Powell (craigpow@uab.edu).

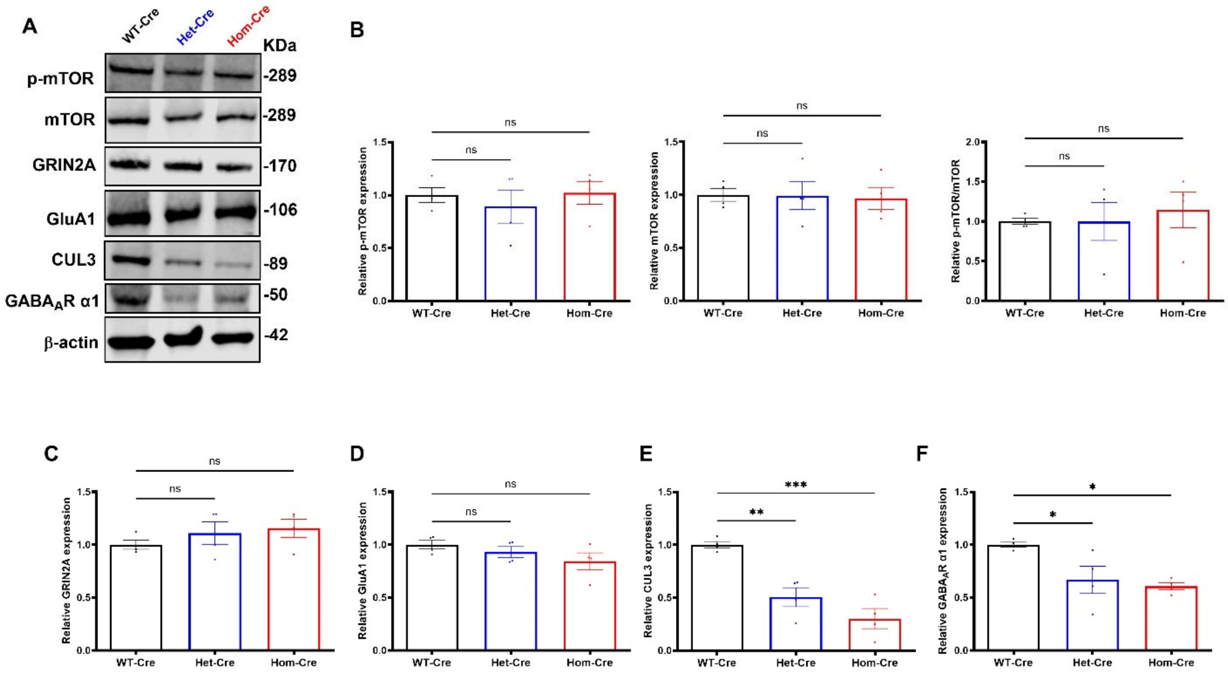

Fig.S1. ***Cul3*** **homozygous deletion and heterozygous reduction decrease $GABA_A$ receptor but not ionotropic glutamate receptor expression in primary cultured hippocampal neurons.**

(A-F) Representative immunoblots (A) and graph of group data (B-F) for anti-p-mTOR, anti-mTOR, anti-GRIN2A, anti-GluA1, anti-CUL3, anti-$GABA_A$R α1 and anti-β-actin blots following AAV-Cre-GFP (WT-Cre, Het-Cre, Hom-Cre) transduction in cultured f*Cul3* hippocampal neurons. *P<0.05, **P<0.01, ***P<0.001, ****P<0.0001, ordinary one-way AVONA with Dunnett's multiple comparisons test; bars represent mean ± SEM, 4 separately derived neuronal cultures.

Data Availability Statement: The data set analyzed in this paper is available at the GIN repository (https://doi.org/10.12751/g-node.bh6pvf).
