# Supplementary figures and images for: Autism risk gene Cul3 alters neuronal morphology via caspase-3 activity in mouse hippocampal neurons
Source: Front Cell Neurosci. 2024 May 9;18:1320784. doi: 10.3389/fncel.2024.1320784 (PMC11129687; doi:10.3389/fncel.2024.1320784)

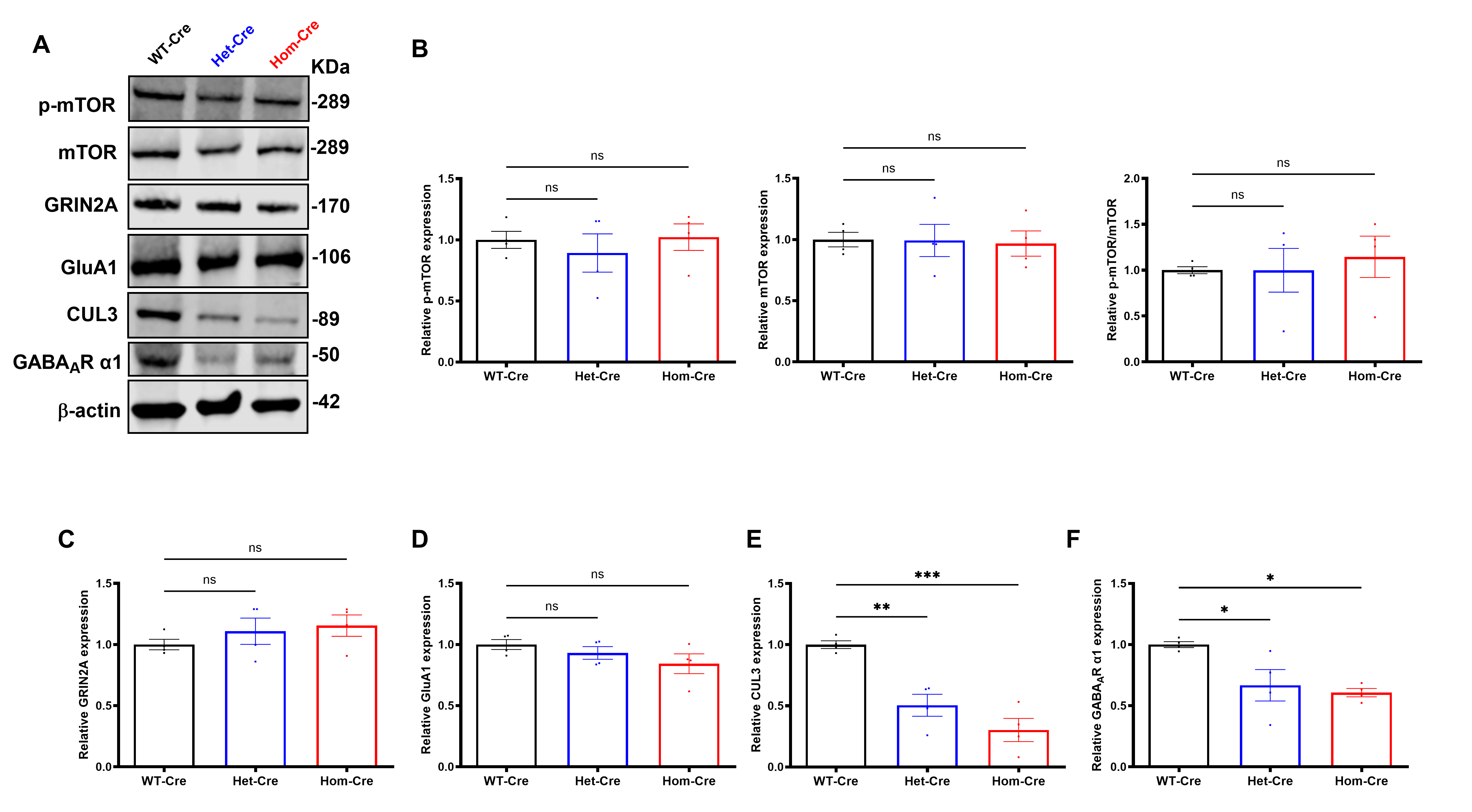

Supplement: Supplementary file 3 [file Image_1.TIF]

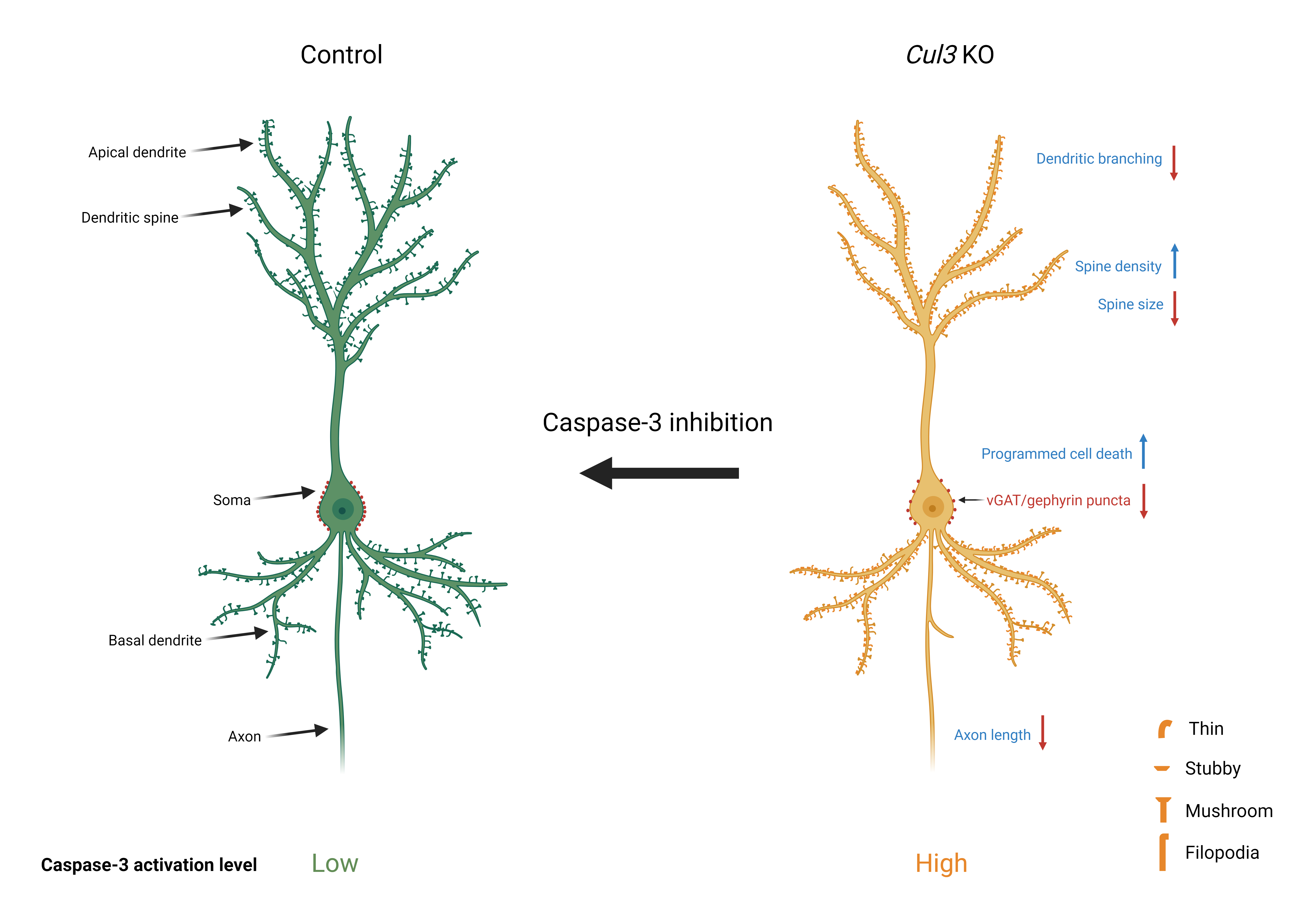

Supplement: Supplementary file 4 [file Image_2.PNG]
